# Understanding the pathways to women’s empowerment in Northern Ghana and the relationship with small-scale irrigation

**DOI:** 10.1007/s10460-021-10291-1

**Published:** 2022-01-17

**Authors:** Elizabeth Bryan, Elisabeth Garner

**Affiliations:** 1 Environment and Production Technology Division, International Food Policy Research Institute, 1201 Eye St., NW, Washington, DC 20005 USA; 2 Cornell University, Ithaca, NY 14850 USA

**Keywords:** Women’s empowerment, Qualitative research, Small-scale irrigation, Ghana

## Abstract

Women’s empowerment is often an important goal of development interventions. This paper explores local perceptions of empowerment in the Upper East Region of Ghana and the pathways through which small-scale irrigation intervention targeted to men and women farmers contributes to women’s empowerment. Using qualitative data collected with 144 farmers and traders through 28 individual interviews and 16 focus group discussions, this paper innovates a framework to integrate the linkages between small-scale irrigation and three dimensions of women’s empowerment: resources, agency, and achievements. The relationship between the components of empowerment and small-scale irrigation are placed within a larger context of social change underlying these relationships. This shows that many women face serious constraints to participating in and benefitting from small-scale irrigation, including difficulties accessing land and water and gender norms that limit women’s ability to control farm assets. Despite these constraints, many women do benefit from participating in irrigated farming activities leading to an increase in their agency and well-being achievements. For some women, these benefits are indirect—these women allocate their time to more preferred activities when the household gains access to modern irrigation technology. The result is a new approach to understanding women’s empowerment in relation to irrigation technology.

## Introduction

Irrigation interventions have considerable potential to contribute to agricultural intensification and farm profitability (You et al. 2011; Giordano et al. 2012; Burney et al. 2013; De Fraiture and Giordano 2014; Giordano and de Fraiture 2014; Xie et al. 2014). Small-scale, farmer-led irrigation is a promising approach to rapidly scale irrigation adoption leading to livelihood and food security gains for vulnerable populations (You et al. 2011; Burney et al. 2013). Moreover, as climate change makes rainfed production riskier, irrigation is emerging as an important strategy to increase resilience to climate shocks and stressors (Nangia and Oweis 2016).

Until recently, less focus has been paid to other potential benefits of irrigation, such as improved nutrition and health, and the pathways through which irrigation contributes to these outcomes. Evidence suggests that small-scale irrigation broadens the range of crops that farmers may cultivate, improves food security and diets (Burney et al. 2010, 2013; Namara et al. 2011; de Fraiture and Giordano 2014; Alaofè et al. 2016; Passarelli et al. 2018), and increases the availability of nutritious foods throughout the year (Baye et al. 2021). Irrigation can also increase economic access to food, asset accumulation, employment opportunities, and spending on education and health care through an income effect (Namara et al. 2011; Burney and Naylor 2012; Passarelli et al. 2018). Several studies have documented that these related benefits, such as improved diets, nutrition, and health, are also associated with women’s empowerment (e.g. Malapit and Quisumbing 2015; Ross et al. 2015). While the literature demonstrates the potential of irrigation to have broad benefits, its direct relationship with women’s empowerment is relatively undocumented.

A growing body of research aims to understanding the potential pathways to support women’s empowerment as an outcome of development interventions (Cornwall 2016; Malapit et al. 2019). One key pathway is through the accumulation of productive assets that provide opportunities for women to earn and control additional income, expand their decision-making authority, and improve their well-being (Kabeer 1999; Meinzen-Dick et al. 2011). A review of eight agricultural development interventions (ranging from gender blind to gender transformative) found that asset levels increased because of the interventions, but only a few led to women’s greater control over assets (Johnson et al. 2016). Importantly, interventions that expand access to assets alone are not enough to support women’s empowerment. According to Cornwall (2016), while external interventions play a role in removing obstacles and creating opportunities for women’s empowerment, women themselves must be the agents of change in their own lives.

Expanding access to agricultural technology and inputs tends to support women’s empowerment, but the evidence remains limited (Anderson et al. 2021). Furthermore, technology adoption can improve outcomes for women, like dietary diversity, and women’s empowerment further magnifies these benefits (Kassie et al. 2020). However, there may be trade-offs with technology adoption, such as labor displacement, that can disproportionately impact women (Vemireddy and Choudhary 2021). Interventions that expand women’s access to technologies for small-scale irrigation, such as motor pumps, therefore, have the potential to support women’s empowerment by expanding their control over agricultural production decisions, income decisions, and time allocation decisions. In some cases, however, irrigation could negatively impact women’s control over land and production as water usage and land values increase, particularly in context of large-scale irrigation projects (Harris 2006). Therefore, there remains a gap in understanding how technologies, like irrigation, foster women’s empowerment and under what conditions.

A gap also exists in understanding how women’s empowerment supports technology adoption and the allocation of benefits from technology use, including in the case of irrigation. For example, an increase in women’s decision-making authority over production and income decisions could lead to the adoption of irrigation systems. Conversely, women’s disempowerment, such as their lack of control over productive assets like land, limited input in household and community decision-making, and heavy workloads often results in lower adoption of irrigation, limited participation in governance of irrigation, or fewer benefits from irrigation (Theis et al. 2018; Imburgia 2019; Lefore et al. 2019). These varied experiences reinforce the need to understand the nuanced and contextual relationship between irrigation and women’s empowerment.

A growing interest in building empirical evidence of interventions’ contributions to women’s empowerment has largely focused on the development of consistent and comparable quantitative measures of empowerment, such as the Women’s Empowerment in Agriculture Index (Alkire et al. 2013; Malapit et al. 2019), with regional (e.g., Southeast Asia, Gupta et al. 2019) and sector adaptations (e.g., for livestock, Galiè et al. 2019). However, quantitative approaches may miss important nuances in local understandings of empowerment and the impact pathways of development interventions that may be uncovered through complementary qualitative research (O’Hara and Clement 2018). This research adapts a well-established conceptual framework of empowerment (Meinzen-Dick et al. 2019, referencing Kabeer 1999) to map the multiple pathways through which a specific small-scale irrigation intervention interacts with aspects of women’s empowerment in the Upper East Region of Ghana. This paper uses qualitative data collected through life history interviews and gender-disaggregated focus groups with men and women farmers and traders. The study centers around an intervention that distributed motor pumps to groups of farmers for irrigation on household plots. The analysis of the relationship between small-scale irrigation and women’s empowerment is placed within an understanding of local definitions of empowerment and underlying processes of social change. The result allows for a systematic analysis of the complex processes of women’s empowerment grounded in theory, while responding to the contextual lived experiences of participants. The results shed light on the ways in which development interventions, particularly those that expand access to small-scale irrigation technologies, interact with women’s empowerment.

## Conceptual framework: linkages between small-scale irrigation and women’s empowerment

**Fig. 1:**
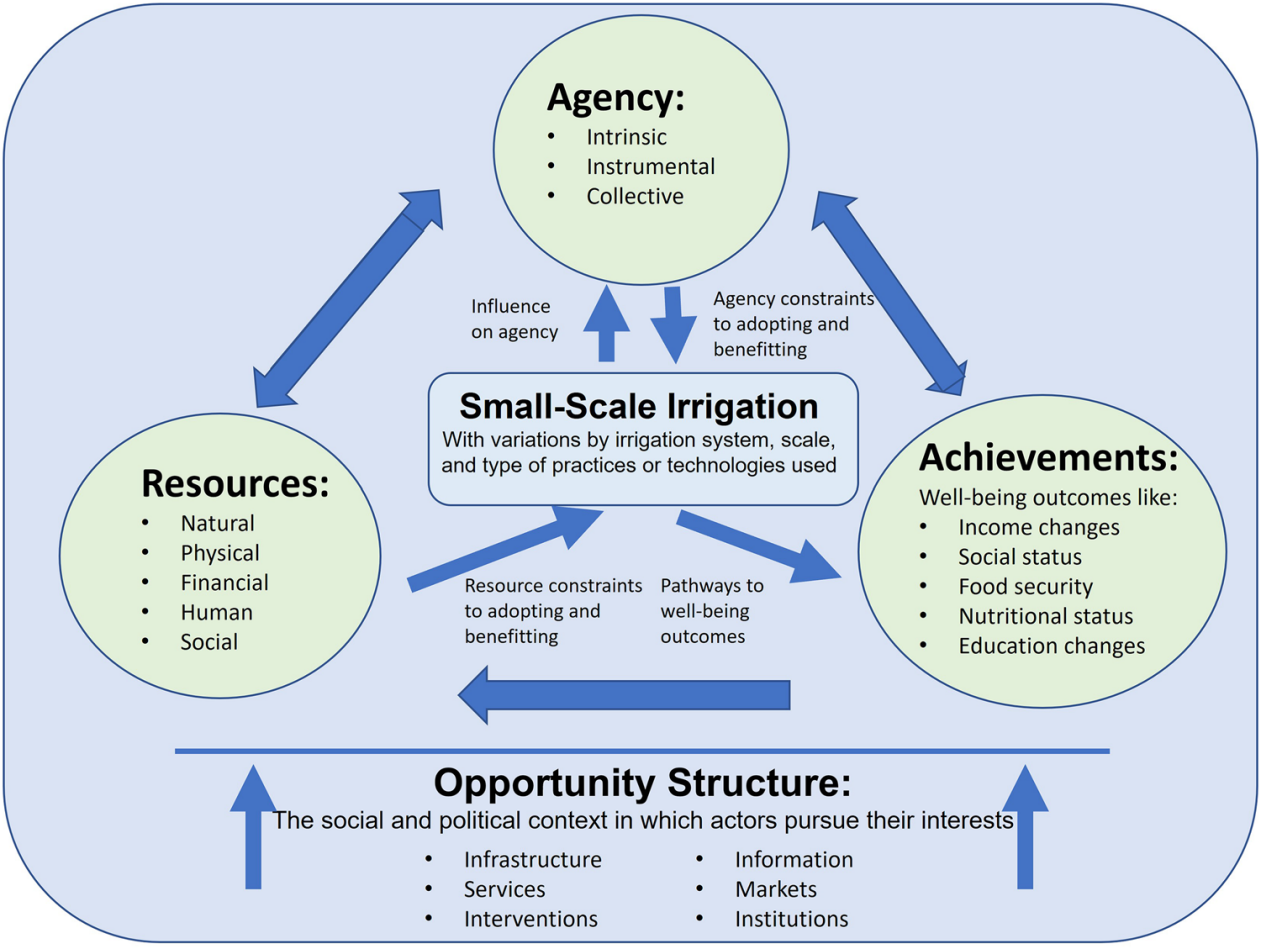
Framework for Small-Scale Irrigation and Women’s Empowerment. *Source* Adapted from Meinzen-Dick et al. (2019), referencing Kabeer 1999)

Women’s empowerment is multi-dimensional and understood as both an outcome (increased access to and control over resources and decision-making ability) and a process of change (the process of expanding people’s freedom to act and capacity to make choices) (Kabeer 1999, 2001; Nussbaum 2000; Datta and Kornberg 2002; Alsop et al. 2006; Stern et al. 2005). The foundational framework for women’s empowerment used for this study draws on the definition developed by Kabeer (1999), as interpreted by Meinzen-Dick et al. (2019). Women’s empowerment in this framework is conceptualized as an iterative process by which individuals improve their ability to make strategic life choices (agency) by utilizing resources, leading to improvements in well-being outcomes (achievements), such as food and nutrition security, and/or economic and social status (Fig. 1).

This study further adapts the women’s empowerment framework of Kabeer (1999) and Meinzen-Dick et al. (2019) to illustrate how the introduction of small-scale irrigation relates to the various components of empowerment. Figure 1 shows that small-scale irrigation interacts with each of the components of empowerment differently. Certain components—namely resources and agency—are needed for women to be able to adopt and utilize small-scale irrigation technologies and practices as shown by the arrows pointing from these components towards small-scale irrigation. Evidence shows that women face greater resource- and agency-related constraints in adopting irrigation practices and technologies, such as less access to land and water for irrigation, less access to financial capital, restrictive social norms, lack of access to knowledge and training, and heavier work burdens at home (van Koppen et al. 2012). These constraints not only limit women’s ability to adopt irrigation practices and technologies but also limit their ability to benefit from them. For example, women have less influence over decisions related to the use of irrigated crops or the spending of income from the sale of irrigated crops (Theis et al. 2018).

At the same time, irrigation interventions may also contribute to women’s agency or disempowerment through changes in their control over agricultural production decisions, income decisions, and time use. Irrigation activities targeted towards women, for example on plots managed by women or on irrigated home gardens, have been shown to increase women’s control over irrigated produce and income, and improve nutritional outcomes (Iannotti et al. 2009; Olney et al. 2009; Burney et al. 2010; van den Bold et al. 2013; Olney et al. 2015). Irrigation can also affect women’s time in different ways. It can either relieve women’s time burden or add to it depending on the type of irrigation technology being applied (e.g., either manual or motor pump). Time allocation may also shift among different family members when technologies are adopted (Theis et al. 2018), which can influence time spent caring for children (Cairncross and Cliff 1987; Burger and Esrey 1995; Miller and Urdinola 2010) or engaging in income-generating activities (Koolwal and Walle 2013).

Finally, irrigation may lead to well-being outcomes (achievements) for women through several pathways (Domènech 2015; Passarelli et al. 2018) and changes in women’s agency intersect these pathways in critical ways. For example, women’s involvement in agricultural and irrigation decisions has implications for production choices. These include the types of crops that are planted, how these crops are used (e.g., sold in the market or consumed at home) (Carr 2008), and how to spend the income earned from selling irrigated crops (Gillespie et al. 2012; Meinzen-Dick et al. 2012). Women’s involvement also has potential positive implications for nutrition, health, and education (Burney et al. 2010; van den Bold et al. 2013).

The resulting relationship between irrigation and women’s empowerment is heavily dependent on the broader social, political, and institutional context that governs men’s and women’s behavior and interactions (the opportunity structure in Fig. 1) (Narayan 2005; Petesch et al. 2005; Alsop et al. 2006). For example, social norms governing men’s and women’s roles in the household and community might prohibit women from engaging in certain activities, like irrigation using manual pumps (Njuki et al. 2014). Women also have different preferences for irrigation practices and technologies given their socially-determined roles in the household, community, and agricultural activities (Theis et al. 2018).

Adapting this framework allows us to understand women’s empowerment, as expressed by women and men of four communities in the Upper East Region of Northern Ghana, in the context of a small-scale irrigation intervention. Rather than relying solely on survey measurements that capture only a moment in time, this framework serves to unpack the complex relationships underlying women’s empowerment and identify the processes through which it is experienced.

## Study area, data, and methods

### Study context

**Fig. 2:**
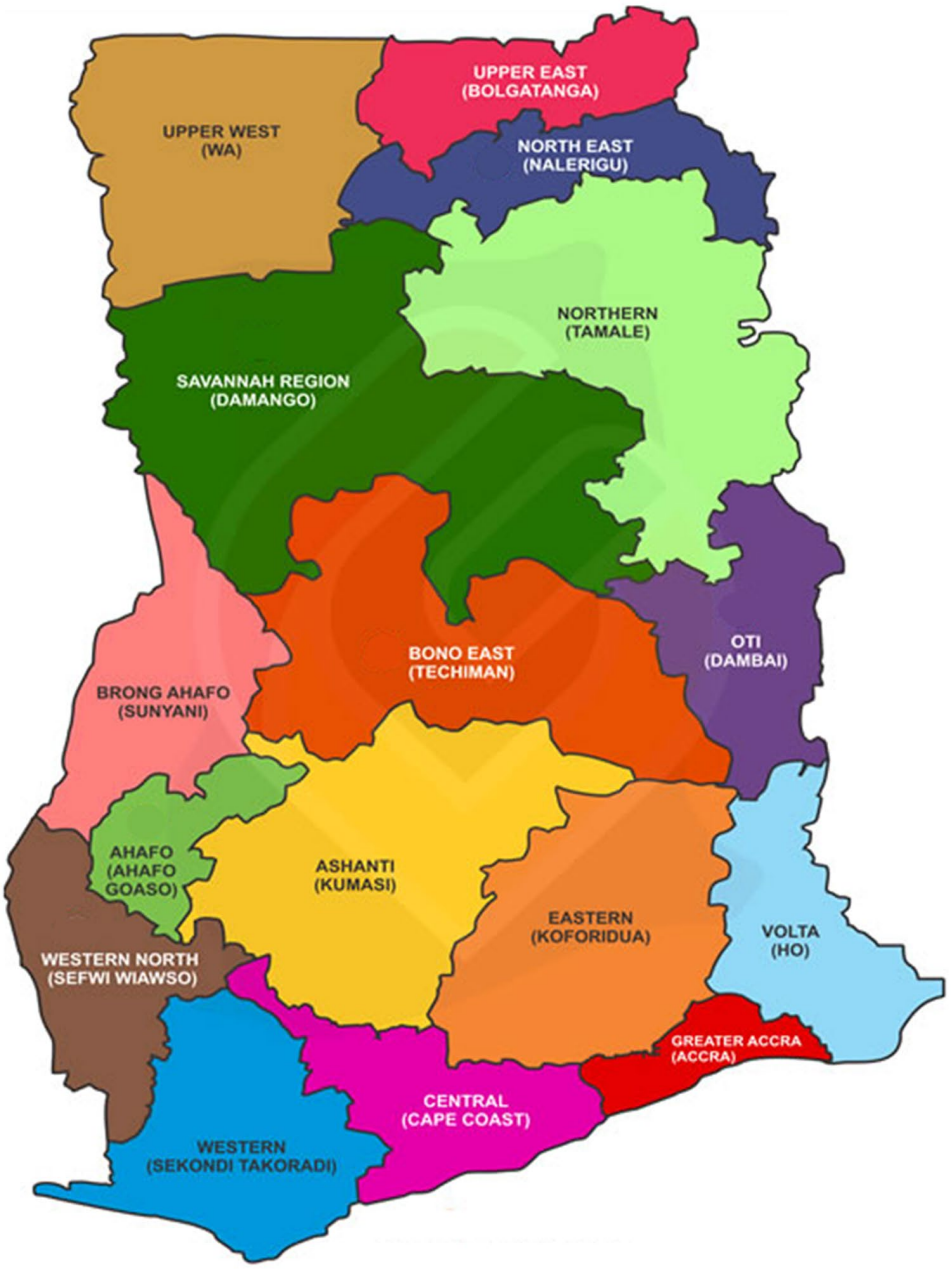
Map of the Regions of Ghana. *Source* The Permanent Mission of Ghana to the United Nations. Note: Approximately one year after data were collected for this study, the Regions of Ghana were revised (December 2018) and 6 new regions were established. This did not affect the designation of the study sites, which remain in the Upper East Region, Garu-Tempane District

The Upper East Region of Ghana, shown in light green at the top right corner of the map in Fig. 2, is characterized by a single rainfall season between May and October, followed by a long dry season, with an annual average rainfall amount of 1000 mm (Ministry of Food and Agriculture 2016; Ampadu and Cudjoe 2020). Production in the region is characterized by rainfed, subsistence production of staple crops, including maize, millet, rice, and soy. Irrigated production takes place mainly during the dry season and is dominated by onions, followed by okra, tomato, red pepper, watermelon, and leafy green vegetables (Mekonnen et al. 2019).

Groundwater is the main source of irrigation water for half the irrigators and is usually obtained by hand dug wells in the riverbed during the dry season, with irrigated plots typically located close to the water source. Small reservoirs (dams) are another main source of irrigation water for about a quarter of households in the study area, providing easier access to water for those with land near the dam. Most irrigating households obtain and apply water using cans or buckets and very few have access to modern technologies for small-scale irrigation, like motor pumps (Mekonnen et al. 2019).

Household survey data collected from the study sites show that many people live in large compounds with their extended family. In 56% of households, men have more than one wife, and 22% of households are female headed. Few household heads (32%) received any schooling and only 15% are literate (18% of male heads are literate, while only 3% of female heads are literate). The average age of male household heads is 53 years while for female heads it is 60 years. Approximately 43% of households are Muslim, while the remaining are Christian (38%) or hold traditional beliefs (19%).

Many households live on the cusp of undernutrition and poverty during the long dry season with few options to improve their well-being, aside from migration to other areas of the country or non-farm employment (Abdulai et al. 2018). The Upper East region experiences the highest incidence of wasting in the country (9%), a level that is considered severe by the World Health Organization (GSS 2015). While calorie availability has increased over time, much of this comes from staple foods (Ecker and Van Asselt 2017) and lack of dietary diversity remains one of the key determinants of child undernutrition in the country (Boah et al. 2019). Small-scale irrigation offers the potential to expand and diversify production to create longer growing seasons that increase nutrient-rich food availability.

### Intervention, sampling, and data collection

Data were collected in July and August 2017 by two researchers (both female) and two facilitators/translators (one female, one male) in four villages and two markets in the Garu-Tempane District of the Upper East Region, Ghana. Selected villages were part of an international development project implemented by iDE. This project operated in nine villages in the Upper East Region of Ghana due to (1) their proximity to ongoing activities and (2) high potential for irrigation based on an ex-ante assessment using biophysical (slope, surface water access, and groundwater access) and socioeconomic (distance to markets) indicators. Within each village, farmers self-organized into groups of five (same-sex and mixed-sex groups) to receive training in group dynamics and micro credit. Villages were randomly divided into treatment (4) and control (5) communities. Within each treatment community, some of the farmer groups were selected to receive a motor pump for small-scale irrigation through a random lottery.

Four villages, two treatment villages (Mongnoori and Yidigu) and two control villages (Akara and Asikiri), were randomly selected for qualitative data collection midway through the intervention. Participants in the research were selected from the set of households that were surveyed between November 2015 and Febrary 2016, before the start of the intervention. Participants were selected to participate in the qualitative research based on their irrigation status at baseline (mix of irrigating and non-irrigating), participation in the program (treatment and control), and individual empowerment scores calculated using the Women’s Empowerment in Agriculture Index (mix of empowered and disempowered) (Alkire et al. 2013).

The qualitative data collection protocols used for the study were adapted from instruments developed for the project-level Women’s Empowerment in Agriculture Index (pro-WEAI) (for details on how these tools were developed see Meinzen-Dick et al. 2019). The two translators/facilitators were trained on the purpose of and methods for implementing the protocols for two days prior to the start of data collection. The protocols were translated during the training following discussions among the research team.

In each village, focus groups on empowerment topics were carried out with two groups of approximately 8 men and women separately with a facilitator/translator of the same sex in the local language. Topics included women’s roles in the community, leadership qualities, participation in community and household decisions, women’s mobility, and inheritance and marital patterns. Two additional focus groups were conducted separately with men and women farmers selected to receive the motor pumps in the two treatment villages. One seasonal calendar focus group (4–5 farmers both men and women) was conducted in each village to identify key gendered livelihood activities and the implications for men’s and women’s time use, income sources and expenditures, and other important events throughout the year. All focus groups lasted around 2 h each.

Life history interviews were carried out with six farmers in each village (two men and four women) for a total of 24 interviews. Each interview lasted between 1 and 1.5 h. In each village interviews covered a mix of irrigators and non-irrigators, pump users and non-pump users, and empowered and disempowered individuals (based on their WEAI score). Women interviewees covered each possible combination of these attributes, while treatment villages only included men irrigators who were empowered and disempowered and men in control villages were non-irrigators (empowered/disempowered). The life history interviews were semi-structured and aimed at giving space for the respondent to discuss the personal experiences that have shaped their attitudes and beliefs. The interviews also explored topics, such as gender roles, decision-making, intra-household dynamics, relationships with the community, and perceptions of self. Interviews were led by two researchers with simultaneous translation by the facilitators/translators.

In addition, four interviews with market traders were carried out: two in a large market (Basyonde) and two in a small market (Garu). Interviews focused on the location of sales and physical access to markets, seasonality, price determination, payment methods, gender barriers to market participation, and market characteristics. Table 1 summarizes participants by data collection method and gender. Interviews lasted for 1–1.5 h and were led by two researchers with simultaneous translation by the facilitators/translators.

**Table 1:** Sample size by method and gender (number of participants)

	Focus groups		Individual interviews		Total participants
	Empowerment topics (same sex) (12 focus groups)	Seasonal calendar (mixed) (4 focus groups)	Life history (24 interviews)	Market trader (4 interviews)	
Female	48	10	16	2	76
Male	48	10	8	2	68
Total	96	20	24	4	144

*Source* Authors

Interviews and focus groups were recorded, transcribed, and translated into English. The transcripts were imported into NVIVO and files were classified according to data collection method, interview, translator, interviewer, and participant data (gender, age of the interviewee, ethnicity, village, irrigation status, pump status, empowerment score). We approached coding as a decision-making process, which considers aspects of the code in line with the methodology and research questions (Elliott 2018). A set of thematic codes (nodes) and sub-themes was developed based on the topics covered in the protocols, which link to the elements in the conceptual framework. Nodes and themes were not considered to be mutually exclusive, and text was coded with multiple nodes or themes where appropriate. Themes that emerged from reading the transcripts were added to the original list of nodes. After coding all transcripts, some nodes or sub-themes were merged or separated to create new nodes. Table 2 presents the list of general nodes used, the description of themes covered within each node, and a description of how these nodes link to elements of the conceptual framework.

**Table 2:** Nodes and sub-themes for qualitative analysis

Nodes: main headings	Description of themes covered	Link to conceptual framework
Income and expenditure decisions	Ability to control income from various livelihood activities and make expenditure decisions in line with personal needs, priorities, and preferences	Indicator of instrumental agency
Intrahousehold relationships	Characterization of the relationships between adult decision-makers in the household (e.g. level interest alignment and cohesion, respect, unity/discord, domestic violence), family structure, marriage and courtship, parenting and parenthood	Indicator of intrinsic, instrumental, and collective agency
Leadership and community	Characteristics of community leaders and powerful people, decision-making processes at the community level, and changes in community leadership roles of men and women over time	Collective agency, enabling environment
Markets	Ability to access markets and participate in market transactions including selling agricultural products and purchasing agricultural inputs or household goods	Enabling environment
Mobility	Ability to travel freely throughout the community, neighboring communities, to local and distant markets, and other important places	Instrumental agency
Nutrition and health	Decisions on food purchases, food preparation, infant and young child feeding practices, medical decisions, health experiences	Achievements
Other decisions	Decisions about other domestic activities (e.g. cleaning, caring for children, fetching water or energy)	Instrumental agency
Crop production	Decisions regarding land allocation, crop choice, planting, division of labor, input use, harvesting, post-harvesting practices, and sale of crops. Access to information regarding crop production	Instrumental agency
Irrigation (achievements, agency and resources)	Experiences with accessing resources for irrigation, decisions related to irrigation at the household and group/community levels, and achievements related to irrigation	Irrigation and the relationship with resources, agency and achievements
Psychological aspects	Aspirations, life satisfaction, self-efficacy, self-esteem	Intrinsic agency
Resources	Inputs to agricultural production and other livelihood activities, productive assets, education and human capital, financial resources, natural resources (land, water, energy), infrastructure	Resources and enabling environment
Shocks	Idiosyncratic shocks (e.g. illness, death of family member), conflict, shocks to production, and climate/weather-related shocks	Achievements
Time	Division of labor, work burden related to domestic work, agricultural production activities, irrigation, other livelihood activities, and overall workload	Instrumental agency

*Source* Authors

## Results

Results from the qualitative data collection are presented on each relationship in the conceptual framework—i.e., between resources and irrigation, agency and irrigation, and achievements and irrigation. While relationships are broken down in the following analysis, the framework emphasizes the continued process and inter-connectedness between these aspects.

### Resources for empowerment and small-scale irrigation linkages

Participants repeatedly mentioned that there are important differences between men and women in terms of their access to and use of resources needed to adopt and sustain irrigation. Women are more constrained in their access to essential natural resources, like land and water, labor, and other agricultural inputs (fertilizer, fencing), which limits their ability to benefit from irrigated production. Men and women are more equally constrained with respect to some resources, like access to enough credit to purchase a motor pump. These differences have considerable implications for the ability of women to adopt and benefit from small-scale irrigation, and their pathway to empowerment.

#### Natural resources

Many farmers in the Upper East Region stressed the importance of having access to land for their success in farming and the ability to provide for their families. Even more than just having access to land, owning land is considered important for empowerment among both men and women famers. However, accessing irrigable land was a much greater challenge for women. Due to patrilineal inheritance systems in Northern Ghana, women primarily access land through their land-owning husbands or by borrowing or renting land from other men in the community or family members living nearby. In most households, depending on the size of the landholding, women are allocated a plot to farm, and in polygynous households, sometimes jointly with their “rival” wives. However, because of population growth, land is becoming increasingly fragmented with sub-divided areas often too small to provide enough food for the family let alone turn a profit. When the land size for the household is too small, women may not be allocated any land to farm for themselves but will contribute unpaid labor to the household plots. When men die, land is mainly passed on to male children. In some cases, widows may still have access to their late husbands’ land through their children, and have more control if children are too young to manage farming operations. In other cases, control of the land may revert to the husband’s older brother or father.

Renting or “begging” for land is common, but there is typically a price to be paid in cash or in kind for access to the land. However, women, whose husbands do not allocate land to them, are often unable to afford to rent land to cultivate for themselves. Some women noted that when they beg for land, they are given lesser quality lands to farm, particularly if they cannot afford to pay to rent better land. Further, when women invest in this land, they risk the owner reclaiming the land once its ability to produce has increased.

[T]he one who has his own land, the land that is fertile, he farms on that. And, if you go to him to beg, he gives you the infertile land. If he is a troublemaker, and you apply fertilizer—after knowing that you did apply fertilizer and the land is now good, he will come for it the next rainy season. So, you would have thought this time it will help you because you applied fertilizer, he has also stopped you from farming there (Yidigu, FGD, women, pump users).

Access to water for irrigation is closely tied with access to land near the water source, which shaped women’s empowerment opportunities and experiences. Communities in the study area access water from ponds or small reservoirs (dams) or groundwater from the dry riverbed. Because there are no conveyance systems, water from these sources is applied to plots located near the source. As irrigable land has higher value, this can magnify the competition over its access. The resulting difficulty for women (and some men) to access land near the water source limits their ability to irrigate.

In Mongnoori, where there is a small reservoir that is well-maintained and more water is available, women reported having better access to water for irrigation. However, even in this community, living farther away from the reservoir made it more difficult for both men and women to access water for irrigation: “We, those who are not strong, cannot travel that distance to work. The people here go to the dams at Basyonde and Zong to farm, which is far. So, we, those who do not have the strength, can’t go there” (Mongnoori, FGD, men, no pump). Women also stressed that traveling to plots far away from the home disadvantaged older women who were unable to make the journey.

In communities where dry season irrigation is done using water from the dry riverbed, accessing water requires digging a hand-dug well each irrigation season—a labor intensive and physically demanding task. Women are not considered physically strong enough to dig the hand-dug wells and rely on their husbands or hired labor to do it. Insufficient water was a constraint for some farmers to benefit from the motor pumps, especially those in Yidigu. According to one man (Yidigu, FGD, pump users), “Water was the challenge [for some people to use the pump…] There were some places you could use the machine. Other places you couldn’t use the machine to get water.”

Lack of water also limits the amount of land people can cultivate during the dry season. As a result, some husbands do not allocate land to their wives, but rather direct them to assist with the irrigated plots that they control. In the case of one man (Yidigu, irrigator), his wives “help” him with the irrigated farming by preparing food for the laborers, picking weeds, and fetching water. Working together on one plot with his wives is more efficient and minimizes the risk if there is not enough water for separate plots: “If we have different plots and we don’t get enough water, it becomes a problem. If we also farm on the same piece of land, and all of us take the jerricans, we can water and finish in no time” (Yidigu, interview, man, irrigator).

#### Financial resources, productive assets, and inputs

Even when women have access to land and water for irrigation, they lack complementary inputs, such as fertilizer or fencing. Women irrigators reported that their lack of access to fertilizer or a delay in the timing of fertilizer application affects the productivity of their irrigated plots, which limits their ability to benefit from small-scale irrigation. Fencing is considered important because it is needed to protect irrigated plots from destruction by livestock during the dry season when they graze freely. As with the hand-dug well, fencing is built by hand each dry season using mud and sticks. Both men and women reported that women do not have the “strength” to build their own fences and do not have the financial resources to hire labor to construct fencing. Because it is a time-consuming and arduous task, men prioritize building fences around their own dry season plots and often do not invest in fencing around their wives’ plots.

Both men and women lack access to financial resources, particularly credit needed to purchase irrigation equipment. Farmers acknowledged that having access to irrigation pumps through the iDE project was helpful for increasing agricultural productivity during the dry season. However, this benefit was not universal, as those who are older, or less physically able, were not able to take advantage of the pumps in the same way. According to one woman focus group participant about receiving the pump, “if we say it didn’t help us, then we are lying. It has helped us a lot. But, our mothers who were not having strength to work in the garden, they wouldn’t know whether it helped or not….” (Mongnoori, FGD, women, pump users).

Some women also reported benefiting directly from engaging in irrigated production, especially when they gained access to motor pumps. The pumps increased these women’s instrumental agency, by providing additional income to expand their independent production activities beyond the small plots allocated to them by their husbands. One woman (Mongnoori, FGD, pump user) mentioned that the pumps “helped us to get money” to rent more land, buy inputs like seeds and fertilizer, and hire labor.

However, not all women have access to motor pumps and, ultimately, their husbands decide how the pumps will be used. In this case, the lack of agency to make decisions over productive resources, makes it more difficult for these assets to contribute to women’s empowerment directly. Moreover, the lack of access to and control over complementary resources, like land, makes pumps an ineffective resource for many. Even those women in groups that received motor pumps said they gave them to their husbands to control, since “[We] can’t do [our] own [irrigated farming] because we don’t have land.” (Yidigu, FGD, women, pump users). Moreover, social norms about ownership of agricultural machinery hindered some women’s ability to benefit from using the pump, even if they themselves participated in groups that gained pump access.

### Women’s agency and small-scale irrigation

The findings indicate that women’s agency is generally increasing in the study areas, irrespective of the irrigation intervention. Participants reported that women are becoming more involved in agricultural production decisions and choosing to engage in other income-earning activities. Women discussed contributing the income they earn to cover household expenses, such as school fees or health expenses; although, for some women this was not considered a positive change. Furthermore, women discussed taking a more active role in the community, including joining groups, with more women becoming respected leaders in the community.

#### Intrinsic agency

Women’s intrinsic agency varies according to individual circumstances, including the level of relative wealth, experience with shocks, and level of education. Overall, the findings suggest that intrinsic agency and achievements are inextricably linked, whereby achievement of personal goals can improve life satisfaction and increase intrinsic motivation. Many women and men expressed the belief that if you were hardworking, you would be successful and achieve your goals. Engaging in irrigation, because it is a labor intensive and difficult activity, contributes to a sense of strength and pride. Men from Yidigu praised women who maintain a dry season garden, describing that others in the community “will see them as proud women” (Yidigu, FGD, men, pump users).

On the other hand, men and women who are not able meet their own basic needs and those who are unable to work due to injury or illness expressed a sense of shame and fatalism about the future. A sense of despair stemmed from some farmers’ inability to improve their welfare despite their best efforts. Lack of intrinsic agency hinders women’s ability to irrigate. Particularly in communities where irrigation is only possible by hand-dug wells, women perceive that they are not physically strong enough to dig a well for irrigation. “We do onion farming, but women are not strong enough to water the onions…. We dig down very deep to fetch water and women can’t dig that deep because they are not strong enough…. I mean they don’t have the energy to dig and fetch water from the pit” (Asikiri, interview, woman, irrigator). While both men and women acknowledge that digging wells is grueling work, the perception that women are not strong enough may also be influenced by cultural norms about gender appropriate work, rather than a lack of strength on the part of the women.

#### Instrumental agency

Participants defined the ability of farmers to exercise instrumental agency by several factors including their ability to participate in and influence production (and other livelihood) decisions, their control over income or participation in spending decisions, and their ability to engage freely in livelihood or social activities that benefit them. Women who are involved in dry season cultivation report direct benefits from irrigation, such as control over income from the irrigated plots they manage, and indirect benefits, including greater income and food security for the household. Access to motor pumps provides even greater benefits by reducing the labor burden of irrigation and increasing income from irrigated production. In many cases, women reported that the introduction of motor pumps freed their time from engaging in irrigated production and allowed them to invest time in other preferred livelihood activities. In this case, an increase in women’s agency from irrigation led to their movement out of irrigated agriculture.

#### Decision-making

There were a range of opinions regarding joint decision-making that often intersected with the age of the respondent and household composition (i.e., the number of wives and the size of the compound). The general trend was towards women participating more in decision-making about important household matters, ranging from production to health care decisions, with younger men and women reporting greater levels of joint decision-making. Many men acknowledged that women play a role in decision-making to accomplish household goals, from providing input to making decisions autonomously.

While men still dominate agricultural decisions, both men and women acknowledged women’s greater participation and input into farming decisions compared to the past. In general, women work on the main rainy season plots, which are predominantly controlled by men, and then also cultivate their own plots of land, which their husbands allocate to them. Work on men’s plots generally takes priority over women’s: “If there is work to be done on his farm, he can say you should come and work there. So, you go and do your own work when his is done” (Asikiri, FGD, women). The prioritization of men’s production activities may also hinder women from investing in the plots they control, including hiring labor to build fences or dig wells for irrigated cultivation.

Women do participate in decisions on the main household plots. Across all villages, most men agreed that taking production decisions jointly is ideal and will have better results (even yields) and they acknowledged that both husbands and wives contribute to the same goals of providing food and income for the family. Despite this recognition, most men and women viewed women’s participation in decision-making as an advisory role while men retain the final say. This was true especially about rainy season production and sale of harvest, which is typically the largest source of household income. Men also tend to make decisions about the output from the household’s main irrigated plots (whether to sell or consume), except in the case of plots allocated for women to manage. For irrigated plots that men manage, women are still responsible for taking the crops to market for sale when directed by their husbands.

There were mixed experiences related to the plots that women cultivate themselves. Some women and men reported that women decide how to manage their own plots and retain control over the income. Others acknowledged that their husbands direct their work on these plots, from deciding which crops should be planted to what is done with crops produced. Some men and women noted that men allocate separate plots to their wives as a risk-mitigating measure. This is because the produce and income earned from men’s and women’s plots is often allocated for different purposes, including saving for unexpected events. In some cases, the food crops women produce are saved as a backup for when the harvest from the main rainfed plots is exhausted or if there is a crop failure. However, men also control productive resources, which limits women’s production options: “A woman cannot just decide that ‘this is what I want to do’ and not tell her husband or landlord…. men are the ones who will release their bullocks to you to plough… Also, you don’t have land to farm so you must inform them” (Asikiri, FGD, women).

While some men favored women’s increased involvement in irrigated production, as well as their financial contribution to the household, some expressed negative opinions about women’s autonomy in dry season production. Interviews suggested that the discomfort is more with women’s control over income, rather than their autonomy in production decisions. “When the women do the work in the dry season, some time ago, the men will transplant the crops, water them and then the women will harvest them and sell them so that the money will be for both of them. But recently, most of the women are wild, that they do not want to do that again. They will plant with their husbands and also they will go out and get another plot and plant for only themselves” (Akara, FGD, men).

#### Control over income

The results of the study show a trend towards women having increased ability to earn their own income and greater control over spending decisions, despite the expressed reluctance of some men. Discussions and interviews revealed considerable variation in the ways in which households make spending decisions and in the degree of knowledge that husbands and wives have about the earnings of their spouse.

While women have greater income-earning opportunities and control over income than they did in the past, this is accompanied by greater expectations about women’s financial contribution to meet household food, education, health, and other basic needs. Women tend to control minor expenditure decisions, like food purchases, whereas other expenditure decisions are made jointly (e.g. paying school fees or seeking medical care). When it comes to large purchases, like motor pumps, men tend to lead the decision but acknowledge that their wives should be informed and consulted as a sign of respect.

Some men supported women earning their own income by allocating land for women to produce crops. The same was also true for irrigated plots that women manage during the dry season: “Yes they [my wives] do have their own plot as well. When my wives and I cultivate eight acres of land we use it for consumption, but I have also given each of them one acre each to cultivate the crops they are interested in and sell their produce for income for themselves” (Mongnoori, interview, man, irrigator). Women also have a stake in spending decisions on income earned from the sale of irrigated crops from plots that their husbands’ control, especially when they have provided labor to produce those crops: “The one who worked on the garden owns the money [from the sale of irrigated crops]” (Mongnoori, FGD, men, pump users). However, as with production, husbands are the final decision-makers on spending decisions.

Women reported having even greater control over income earned through other livelihood activities, like trading, pito brewing (local alcoholic beverage), shea butter processing, dawadawa (local spice) making, providing services, and fuelwood production. Some women prefer earning their income through these activities, rather than sharing income earned through farming with their husbands: “I would prefer hair dressing. If it is hair dressing, the proceeds would be for me but with the sale of the pepper, I will share with my husband.… We both take care of the pepper and when it is matured, we harvest and I sell them” (Basyonde, market trader, interview).

Women’s increasing earnings and financial contribution changes power dynamics in the home. Some men seemed relieved that women can reduce their financial burden, and some expected women to cover household expenditures like school fees and food. A woman from Akara noted that she is able to earn income for herself only because her husband does not earn enough: “If I am going to do labor work, or trade or anything, it is because he [husband] doesn’t have enough to support me and, therefore, he will allow me to do it. If he had money to give me, he would have prevented me from doing this work” (Akara, interview, woman, irrigator). Some men considered the income their wives earned as their own, while others were uncomfortable with women’s increasing contribution and see it as a threat to their role as household head and provider. “[If a woman has more money than the man] in our Kusaug tradition, there will be a problem. When you talk, she will not mind you; and if you do not take care, she can even beat you” (Mongnoori, men, FGD, no pump).

Women also had mixed feelings about growing expectations for their contribution. Some felt that it was a burden to have the responsibility to bring in income when they do not have the means to earn enough. Others wanted more independence and felt pride in their ability to provide for their family. One woman from Akara described the pressure placed on her to provide for her family: “My father farmed a lot and had large stock of food and so looking for food to feed the family was not the job of my mothers and my father also supported his children's education. But now everything is on me. My husband is not able to support” (Akara, interview, woman, irrigator).

#### Time burden

Women in the study area have a heavy workload with domestic responsibilities, farming, and other livelihood activities. Irrigated production takes place during the dry season (lasting 4 months) and women play a large role in watering crops using traditional, labor-intensive methods. Because crops must be watered continuously during this period, irrigated production prevents people from engaging in other activities or traveling to visit family in other communities.

Depending on the source of water (dam or dug well), the location of the plot with respect to the water source, and the irrigation technology available, the time it takes to irrigate varies dramatically. Both men and women farmers who use traditional methods view irrigation as a physically exhausting activity. While many women engage in irrigation using traditional methods, especially women whose husbands are unable, many men considered it as sparing their wives if they don’t have to engage in irrigated production: “My wife can help in the garden, especially in transplanting. But if it is watering the plants, women because they carry children or are pregnant, it is dangerous to go to the well and back, so I don’t allow my wife to help. I do most of this myself” (Akara, interview, man, irrigator).

Even though it is tedious, some women want to have the opportunity to engage in dry season farming because of the benefits, like having access to vegetables, and prefer irrigation to other dry season activities, like burning charcoal. While irrigating with traditional methods is considered burdensome, having access to pumps saves women’s time watering or allows them to leave irrigating to their husbands. “Your husband farms [in gardens] and you water and thank God associations have come and we can now get access to machines [pumps] and the men will use them to irrigate. So, now we only observe, and they irrigate” (Mongnoori, FGD, women, pump users).

#### Collective agency

Both men and women placed considerable value on collective agency, whether it be through working together as a family unit or participating in groups at the community level. Working together enables women to make strategic choices, leading to better well-being outcomes or achievements. Women are also increasingly involved in groups which facilitate their access to financial resources (especially through shared savings groups), information and training, and resources from outside groups, like NGOs. However, even when women participate in same-sex groups, group activities sometimes require the approval of husbands. As women in Akara reported, “If I see that an organization like that comes to help us with our water issues, I have to talk to my husband first and tell him of the benefit of the proposed activities of the organization and we will then decide” (Akara, FGD, women). Some women focus group participants also described participating in joint farming activities on rented plots of land with some success. However, the landowner reclaimed land after the first season. Thus, resource constraints related to land access trumped women’s collective effort to gain greater autonomy in production.

Collective agency facilitates the purchase, use, and maintenance of irrigation equipment. This is because purchasing and maintaining pumps is costly and difficult for individual farmers or farm households. Arrangements for sharing or renting modern irrigation equipment are not generally available. Participants reported that the groups formed through the project facilitated labor sharing: “He will come with the bicycle and take it [the pump] and ask you to help him on his farm. We helped each other. If one person is going to do it, one has to help him, when it comes to you too, he will help you” (Yidigu, FGD, men, pump users). However, sharing the pump was sometimes difficult, particularly when farmers in the same group were not located near each other. Men in Mongnoori indicated that moving the pump between group members was even more difficult for women.

### Well-being achievements and small-scale irrigation

Personal achievements mentioned by study participants include economic status, being able to meet basic needs, education, success in farming, and maintaining food security—all of which are strongly linked and contribute to one’s social status in the community. Leaders in the community were viewed as having a higher level of financial security, education, social connections, and leadership qualities that enabled them to offer help to others. Such descriptions provide an understanding of what members of the community can aspire to achieve. In both cases, at the personal or family level as well as at the community level, many of the achievements people described related to doing things for others. For example, many men and women discussed goals related to educating their children rather than themselves.

Engaging in irrigated production increases the social status of both men and women as it demonstrates that a person is hardworking. In particular, women who do dry season production are respected by both men and women in the community as hardworking contributors to their family’s well-being. Women in the focus group in Akara described irrigators as “good women.” Men also value the contribution of these “hardworking” women: “They [women who do dry season garden work] are women who are hardworking…and look beautiful. The women who are not working in the garden they are not like them” (Mongnoori, FGD, men, no pump).

Irrigation also provides resources for families to afford school fees and medical expenses, which support health outcomes as achievements: “Some [crops irrigated with pumps] were sold and others consumed. Part of the money [from the sale of irrigated crops] was used to pay school fees and your child might not feel well, and you could send him or her to the hospital” (Mongnoori, FGD, men, pump users).

Irrigation brings greater food security by increasing the stability of food supply over the course of the year: “If you don’t work in the garden, you will sell the food crops you harvested during the rainy and you will be in hunger” (Mongnoori, FGD, men, pump users). Households also produce different crops with irrigation and consume a portion of what they grow leading to an improvement in diet quality: “We farmed different crops [when we got the pump], we plant onion, tomatoes, pepper, okra, garden eggs, and vegetables” (Yidigu, FGD, men, pump users).

## Discussion and conclusions

Given increasing interest in women’s empowerment by development organizations, it is important to consider the ways in which interventions may overlay theoretical and conceptual understandings of empowerment. The irrigation project in the Upper East Region of Northern Ghana provides an example of how the three dimensions of women’s empowerment (resources, agency, and achievements) can be influenced by irrigation interventions and the extent to which irrigation offers a pathway to women’s empowerment. These lessons hold significant opportunities for both deepening our understanding of women’s experiences as well as informing future interventions.

This paper contributes to the growing body of literature and interest in understanding the opportunity for interventions to support women’s empowerment as an outcome. Efforts are being made to develop coordinated and consistent tools and methods to measure changes in women’s empowerment outcomes across contexts, using both qualitative and quantitative approaches that are linked to widely accepted theoretical concepts of empowerment (Malapit et al. 2019). While the need for consistent measurement is important, qualitative research offers the opportunity to explore local understanding of empowerment in ways that both challenge and validate academic concepts (Meinzen-Dick et al. 2019). For example, while researchers often focus on measures of women’s agency as a key component of empowerment, qualitative research across different cultures and local contexts found that women tend to place greater emphasis on achievements, such as increasing income and helping others, rather than agency (Meinzen-Dick et al. 2019).

While the framework adapted in this paper provides a structure for understanding the pathways for women’s empowerment through irrigation, there are still limitations to its application. In a few cases, there were central themes that shaped women’s ability to adopt and benefit from irrigation in a way that fostered empowerment that were not limited to a particular empowerment dimension. For example, “strength” was often mentioned by participants as a critical component for irrigating successfully. However, this term often conflated physical strength (resources), financial capacity (resources), and willingness to participate in the activity (agency), in ways that were both real (achievements) and perceived, given social norms about labor allocation (opportunity structure). In this case, the framework is limited in how to analyze this concept’s impact on empowerment as it is split across several dimensions. In general, however, it was possible to analyze the themes that emerged using the theoretical constructs of resources, agency, and achievements, even though the research participants themselves did not make such distinctions.

Evidence of the potential for irrigation to contribute to women’s empowerment tends to be scattered across context-specific case studies without a unifying framework for tracing the interaction between irrigation and empowerment and the conditions under which irrigation contributes to women’s empowerment (Bryan and Lefore 2021). This literature suggests that the ways in which irrigation influences women’s empowerment depends on contextual factors (the opportunity structure) as well as the type of irrigation technology, the scale of the irrigation system, and the approach used to implement the intervention (Bryan and Lefore 2021). Thus, when applied to other contexts and interventions, the framework adapted in this paper may yield quite different results. Some of the relationships between irrigation and empowerment observed in this context as a result of the motor pump intervention may play out similarly in other contexts. However, it would not be appropriate to draw general conclusions from the application of the framework in this study.

That said, the results of this research provide several insights about the potential pathways for small-scale irrigation to influence women’s empowerment. The findings showed that women did not necessarily benefit from irrigation in direct ways, such as through control over the motor pumps as a productive asset, but rather through more indirect ways, such as reduced labor burden in agriculture, particularly in irrigated production. Social norms typically prohibit women from owning large assets, like livestock, land, or pumps, as has been demonstrated by other research in this context (Doss et al. 2014; Lambrecht 2016). Even when women acquired motor pumps through groups, they considered their husband to have control over the asset. However, when motor pumps became available, many women viewed the decreased time burden as an opportunity to focus on more preferred livelihood activities.

The results also pointed to serious resource constraints that limit the extent to which women can participate in irrigated production. Specifically, women can only access land for irrigated cultivation through their husbands and, therefore, have less control over the decision of whether to produce irrigated crops. Another study from Northern Ghana similarly shows that landownership strongly affects aspects of women’s agency and achievements, including participation in agricultural decision-making, decisions on farm income, group membership, and time allocation (Yokying and Lambrecht 2020). In other contexts, such as India, women have overcome resource constraints by collectively cultivating leased land with other women farmers (Agarwal 2020). In Northern Ghana, women’s collective agency is growing, including through other economic activities outside of agriculture. However, given the difficulties some groups of women faced in maintaining access to leased land for autonomous production, this approach may be more difficult in the study context without specific outside intervention to facilitate and maintain women’s land access. Thus, it is not necessarily the lack of collective mobilization, but the challenge of access to resources despite collective action that hinders women from benefitting fully from irrigated production.

Resource constraints were even greater for women in villages where water scarcity concerns were more prominent. These challenges will only intensify as pressure due to population growth leads to greater land fragmentation and natural resource scarcity (Abubakari et al. 2016). It is not surprising then, that a framed field experiment by Kramer and Lambrect (2019) found that women in Northern Ghana prefer to allocate resources towards business activities, like trading agricultural and non-agricultural products and food processing, and that both men and women value diversified investments. For some women in our study, it is also the perceived opportunities to directly benefit from engaging in alternative livelihood activities that lead them to pursue other pathways to empowerment outside of agriculture. While some women are drawn to alternative income-earning opportunities, women that do engage in irrigation reported directly benefiting from this activity, including through control over income from the plots they cultivate themselves. This finding has also been observed in other contexts, such as Ethiopia, Tanzania, and Kenya, especially when women’s irrigated production is limited to smaller sales of vegetables (Theis et al. 2018) or to lower value crops (Njuki et al. 2014).

To the extent that engaging in irrigation increases women’s control over income and contribution to household expenditures, this may also indirectly increase women’s decision-making role in the household. However, other studies have shown that economic interventions alone are not sufficient to dramatically increase women’s decision-making authority. To achieve women’s empowerment and gender equality, programs must integrate gender transformative approaches, such as facilitated household dialogues (e.g. Karimli et al. 2021).

The framework developed in this study to understand women’s empowerment pathways could be applied in other contexts and to other types of irrigation interventions. Doing so would inform the ways in which interventions are designed, introduced, and implemented to ensure that they address gender-specific constraints and consider women’s preferences. Such analyses are especially important as opportunities for irrigated cultivation expand, through investments in irrigation infrastructure and the dissemination of irrigation technologies, to ensure that women participate in and benefit from these interventions.

## Abbreviations

WEAI: Women’s Empowerment in Agriculture Index
FGDs: Focus group discussions
